# Neuroprotection of photoreceptors by combined inhibition of both Fas and autophagy pathways in P23H mice

**DOI:** 10.1038/s41419-025-07793-9

**Published:** 2025-07-01

**Authors:** Mengling Yang, Jingyu Yao, Lin Jia, Andrew J. Kocab, David N. Zacks

**Affiliations:** 1 Department of Ophthalmology and Visual Sciences, University of Michigan, Kellogg Eye Center, Ann Arbor, MI USA; 2 Department of Ophthalmology, Xiangya Hospital, Xiangya School of Medicine, Central South University, Changsha, Hunan China; 3 ONL Therapeutics Inc., Ann Arbor, MI USA

**Keywords:** Cell death, Apoptosis, Autophagy

## Abstract

The P23H variant of rhodopsin (RHO) is a common cause of autosomal dominant retinitis pigmentosa (adRP). Our previous data have shown that both the Fas (CD95) death receptor and hyperactivation of autophagy contribute to photoreceptor (PR) death in a mouse model of P23H-RHO adRP. Individually, inhibition of Fas or suppression of autophagy flux improves PR survival and function. The purpose of this study is to examine whether combined inhibition of Fas receptor activation and reducing autophagy flux would have an additive effect on PR survival and function in the P23H mouse. We crossed the Lpr mouse (which contains a functional knockout of the Fas receptor) with the P23H mouse to generate the Lpr/P23H mouse. Hydroxychloroquine (HCQ) was given in the drinking water at P21 to reduce autophagy flux. As an alternative to genetic inhibition of the Fas receptor, pharmacological blockade of the Fas receptor was achieved using intravitreal injections of the Fas inhibitor, ONL1204, administered via intravitreal injection at P14 and 2 months of age. Fellow eyes were injected with vehicle solution as controls. PR cell death, structure and function of the retina, as well as the activation of immune cells, were evaluated. Consistent with previous data, the Lpr/P23H mice exhibited a decreased rate of photoreceptor degeneration and reduced inflammation compared with P23H. Treatment of these mice with HCQ further preserved photoreceptor survival and function lowered the activation of immune cells, and resulted in reduced production of inflammatory cytokines in the retina. These results were recapitulated in HCQ-treated P23H mice receiving intravitreal injections of ONL1204. Our data suggest that in the mouse model of P23H adRD, inhibition of both the Fas pathway and autophagy pathways results in a greater protective effect, demonstrating the potential multipronged therapeutic approach to reduce PR death and improve retinal function in patients with P23H.

## Introduction

Inherited retinal degeneration (IRD) results from mutations in any one of nearly 300 different genes, leading to progressive photoreceptor (PR) cell death and vision loss [1]. With a prevalence of ~1 in 1380 in the United States [2], IRD is one of the most common hereditary retinal diseases [3]. The extreme genetic heterogeneity limits the development of targeted treatments for specific mutations. Therefore, it is crucial to develop treatment methods targeting widely shared pathophysiological pathways, such as cell death and inflammation, that lead to retinal degeneration.

Mutations in the gene encoding rhodopsin (RHO) are a common cause of autosomal dominant retinitis pigmentosa (adRP) in the United States [4]. The Pro23His variant of RHO, resulting from the substitution of histidine for proline at amino acid residue 23 of rhodopsin (RHO^P23H^; herein referred to as P23H), leads to misfolding of rhodopsin [4–7], endoplasmic reticulum (ER) stress, and the activation of multiple cell death pathways, including autophagy, apoptosis, and necroptosis [8–21]. Our previous data in a mouse model of P23H have shown that misfolded rhodopsin results in persistent activation of autophagy and secondary proteasome insufficiency, which in turn contributes to PR death [11, 12]. We have demonstrated that decreasing autophagy in the P23H mice by treatment with hydroxychloroquine (HCQ), an inhibitor of autophagy flux, reduces retinal degeneration [11].

Our previous work in the P23H mouse showed that the Fas receptor is also activated in the retina and contributes to PR death. Fas (CD95) is a well-studied death receptor, and its binding to Fas Ligand (FasL) activates the caspase cascade to induce cell death [22]. Fas also plays a critical role in mediating inflammation. Fas has shown promise as a therapeutic target for photoreceptor protection in a variety of ocular disease models [23–31]. We have previously demonstrated that crossing P23H mice with a Fas-deficient mouse known as the Lpr mouse (cross heretofore referred to as the Lpr/P23H mouse) results in PR protection. Our recent work has also shown the protective effect of pharmacological inhibition of the Fas pathway in the P23H mouse retina by intravitreal injections of ONL1204, a small peptide inhibitor of the Fas receptor [32]. This peptide is based on studies by Zou and colleagues, who showed that the extracellular N-terminus of the alpha-subunit of the hepatocyte growth factor receptor encoded by the *MET* gene is necessary and sufficient to bind the extracellular portion of Fas and to act as a FasL antagonist [33]. Aside from the protective effect of Fas inhibition in the P23H mouse model, we have previously demonstrated the PR-protective effect of Fas inhibition in mouse models of retinal detachment and age-related macular degeneration (AMD), and a beneficial effect on retinal ganglion cell survival in a mouse microbead model of glaucoma [23, 28, 30]. We have also shown that Fas inhibition can protect PR cells in the rd10 mouse, a model of autosomal recessive retinitis pigmentosa [32].

Given the individual protective effect of Fas inhibition and suppression of autophagy flux in P23H mice [11, 31], we sought to investigate whether combining these two approaches would have an additive effect on PR survival. We hypothesize that combining autophagy inhibition using HCQ with Fas inhibition, either genetic or pharmacologic, would provide improved PR protection compared to either approach alone.

## Materials and methods

### Animals and treatments

All experiments were performed following the Association for Research in Vision and Ophthalmology statement for the ethical use of animals and were approved by the University Committee on Use and Care of Animals at the University of Michigan. The Rho^P23H/P23H^ mice were crossed with Fas-Lpr (referred to as Lpr in this study) mice to produce Lpr/P23H mice. All the strains were purchased from Jackson lab (strain # 000485 [Fas-lpr] and 017628 [P23H]) and were on the C57BL/6J genetic background. For experiments with P23H mice, only those heterozygous for the P23H allele were used, as this represents the more common clinical presentation. All mice used in this study were negative for mutation in the Crb1rd8 gene. Mice were housed under standard 12 h of light: 12 h of dark. For experiments with HCQ, the HCQ was administered through drinking water at a concentration of 1.2 g/mL starting at weaning (postnatal day 21), while normal drinking water was given to the control group. For experiments involving ONL1204 administration, this was administered in a 1 µL volume via intravitreal injection to the left eye, with the fellow eye receiving a vehicle solution as a control. Two intravitreal injections were performed—the first at P14 and the second at 2 months of age.

### Antibodies

Primary antibodies used in this study were: RHO (4D2, Novus Biologicals, NBP1-48334; 1:2000), m-Opsin (Millipore, AB-5405,1:1000), Iba1 (Novus Biologicals, NB100-1028, 1:100); Secondary antibodies are from Invitrogen (Paisley, UK): goat anti-mouse Alexa Fluor 546 (1:1000, A-11030), goat anti-rabbit Alex Fluor 488 (1:1000, A-10008) and donkey anti-goat Alexa Fluor 488 (1:1000, A-11055).

### Histology

Eyes were enucleated after marking the cornea at the 12 o’clock position for orientation, then fixed in 4% paraformaldehyde overnight at 4 °C before paraffin embedding. 6 µm paraffin sections were produced using a microtome (Shandon AS325, Thermo Scientific, Cheshire, England, UK). Hematoxylin (Fisher Scientific, Hercules, CA, USA) and eosin (Fisher Scientific) staining was performed as described previously [34]. Only sections crossing the optic nerve, containing both the superior and inferior aspects of the retina were used for staining and images were obtained with a Leica DM6000 microscope (Leica Corp., Wetzlar, Germany).

### Immunofluorescence on retinal sections

The cornea was marked for orientation and retinal cryo-sections were prepared as described previously [34]. Sections were blocked with 5% BSA with 0.3% Triton X-100 (PBST; Sigma-Aldrich) for 1 h followed by 3 washes with PBST. Sections were incubated with primary antibodies at 4 °C overnight. After being washed with PBST, the sections were incubated with secondary antibodies at room temperature for 1 h. ProLong Gold with DAPI (Invitrogen, P36941) was applied to mount the slides. Only sections crossing the optic nerve, containing the superior and inferior aspects of the retina were used for staining, and images were taken at comparable areas (600 µm from the optic nerve in the inferior retina) of the sections with a fixed gain using a confocal microscope (Leica STELLARIS 8 FALCON Confocal Microscope., Germany). In this study, 6 µm paraffin sections were used for RHO and m-Opsin staining, and 30 µm cryo-sections were used for Iba1 staining.

### Immunofluorescence on retinal whole mount

Preparation of the retinas was performed as described previously [34]. Retina samples were incubated with Iba1 antibody at 4 °C for 48 h. After washing with 0.3% Triton PBS, the retinas were incubated with secondary antibodies overnight at 4 °C followed by 3 more washes. Retinas were then incubated with DAPI at room temperature for 2 h and then mounted on a glass slide (Fisher Scientific, Pittsburgh, PA, USA) with the ganglion cell layer facing upward. Z-section confocal images of comparable areas of the superior and inferior retina were taken with a Leica STELLARIS 8 FALCON Confocal Microscope (Leica Corp., Wetzlar, Germany).

### Terminal deoxynucleotidyl transferase dUTP nick end labeling

Terminal deoxynucleotidyl transferase dUTP nick end labeling (TUNEL) was performed on 10 µm cryo-sections crossing the optic nerve. For HCQ-treated Lpr/P23H, untreated Lpr/P23H, and their C57 controls, the eyes were sampled at age P30. TUNEL was performed using the DeadEnd Colorimetric TUNEL System (Promega Corporation, Madison, WI, USA) according to the manufacturer’s instructions. For samples of P23H mice, 5 nonoverlapping sections of each sample were used, and images were taken at ×20 magnification for each section. The total number of TUNEL-positive photoreceptors in the whole section was counted and averaged for each sample. The number of TUNEL positive photoreceptors in each ×20 magnification image was counted and averaged for each sample.

### Caspase 8 activity assay

Caspase 8 activity assays were performed as previously described [34]. Two to three retinas of left eyes (HCQ + ONL1204 injection) or right eyes (HCQ + Vehicle injection) from 2 to 3 mice were pooled as one sample, then homogenized in a lysis buffer (20 mM MOPS, pH 7.0, 2 mM EGTA, 5 mM EDTA, and 0.1% Triton X-100) with the presence of protease inhibitor (Complete Protease Inhibitor Tablet [11697498001; Roche, Indianapolis, IN, USA]). Samples were centrifuged at 10,000 × *g* for a 1 min at 4 °C. Subsequently, each assay utilized 150 µg of protein. Samples were loaded in duplicate and Caspase 8 activity was analyzed using the Caspase-8 Activity Assay Kit (Colorimetric) (NBP2-54817, Novus Biologicals) with a plate reader. The values of caspase 8 activity were then normalized to the ONL thickness of the retina as measured by OCT.

### Real-time polymerase chain reaction (RT-PCR)

A purification kit (Qiagen, 74104) was used for the isolation of RNA from one retina of each mouse. 500 ng of total RNA was converted into cDNA with the SuperScript III Reverse Transcriptionase Kit (18080093; Thermo Fisher Scientific). Transcript levels were assayed in triplicate using a thermal cycler (Bio-Rad CFX96 Real-Time System, C1000 Touch Thermal Cycler; Bio-Rad Laboratories, Hercules, CA, USA). Target gene expression levels were normalized to the level of Rpl19 using a comparative Ct method. Specific primers were as follows: Rpl19 (forward 5′-ATGCCAACTCCCGTCAGCAG-3′; reverse 5′-TCATCCTTCTCATCCAGGTCACC-3′). Ccl2 (forward 5′-CGTTAACTGCATCTGGCTGA-3′, reverse 5′-AGCACCAGC-CAACTCTCACT-3′). The PCR cycling conditions consisted of an initial denaturation of 95 °C for 10 min followed by 40 cycles of 95 °C for 15–30 s and 60 °C for 1 min.

### Optical coherence tomography

Optical coherence tomography (OCT) was conducted as previously described [11] using the spectral domain OCT system from Bioptigen, Inc. (Durham, NC, USA). The outer nuclear layer (ONL) thickness was measured at 250 and 500 µm superiorly and inferiorly from the optic nerve for both P23H and Lpr/P23H groups.

### Electroretinography

Electroretinography (ERG) was conducted utilizing the Espion e2 recording system (Diagnosys, Lowell, MA, USA) as previously documented [11]. Following an overnight period of dark adaptation, scotopic ERG measurements were taken at 0.01, 10, and 32 log cd s/m². After 10 min of light adaptation, the photopic function was evaluated at 10, 32, and 100 log cd s/m². The resulting amplitudes were analyzed with Espion V6 software (Diagnosys, Lowell, MA, USA).

### Statistical analysis

Student’s *t*-test was used to compare data between the two groups. To compare ONL1204-injected eyes with fellow eyes injected with the vehicle, a one-tailed Wilcoxon test was utilized. For evaluations across the three groups, a 1-way ANOVA was utilized, followed by a Tukey multiple comparison test. The statistical analysis and graphing were performed using Prism (GraphPad, Inc., La Jolla, CA, USA) and Microsoft Office Excel (Richmond, WA, USA). Data is expressed as mean ± standard deviation. Differences were considered significant at *p* < 0.05.

## Results

### HCQ confers increased PR protection in the Lpr/P23H mouse retina

To examine whether combined inhibition of both autophagy and Fas pathways could further protect photoreceptor survival, we first conducted a proof-of-concept study in the Lpr/P23H mice, which were generated by crossing P23H mice with Fas-lpr mice. The Fas-lpr mouse line contains a mutation in the Fas receptor that renders it inactive [35]. We have previously shown that in Lpr/P23H, the absence of Fas activity reduced photoreceptor cell death in the P23H retina as compared to the P23H mice [31]. We assessed whether reducing autophagy flux with HCQ treatment can further increase photoreceptor survival in the Lpr/P23H mice.

**Fig. 1:**
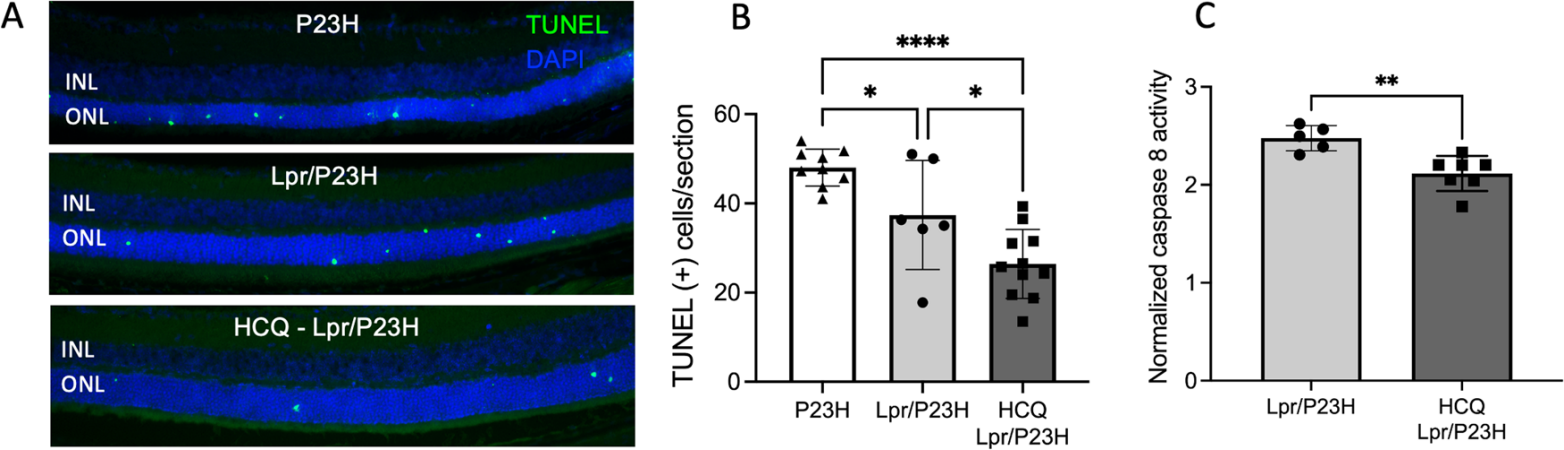
Decreased photoreceptor cell death and inflammation in the HCQ-treated Lpr/P23H mice. **A** Representative TUNEL staining images and **B** quantification of TUNEL-positive cells in the ONL for P23H, Lpr/P23H mice, and HCQ-Lpr/P23H mice at P30 (*n* = 9 for P23H; *n* = 6 for Lpr/P23H and *n* = 11 for HCQ-Lpr/P23H. One-way ANOVA.). **C** Quantification of caspase 8 activity at 1 month of age (*n* = 7 for HCQ-treated Lpr/P23H, *n* = 6 for untreated Lpr/P23H). *P < 0.05, **P < 0.01; ****P < 0.0001. *t*-test. INL inner nuclear layer, ONL outer nuclear layer.

HCQ was provided in the drinking water starting at age P21 once the mice were weaned. At 1 month of age, consistent with our previous work, the Lpr/P23H mice exhibited a lower number of photoreceptors undergoing apoptosis, as indicated by TUNEL staining in the outer nuclear layer (ONL), compared to P23H controls. The HCQ-treated Lpr/P23H mice showed a further significant reduction in the number of TUNEL-positive cells compared with untreated Lpr/P23H mice (Fig. 1A and B). Caspase 8 is considered the first downstream target of the activated Fas receptor and a hallmark of Fas activation [36]. In the retinas of the Lpr/P23H mice treated with HCQ, caspase 8 activity was decreased as compared to untreated Lpr/P23H mice (Fig. 1C).

**Fig. 2:**
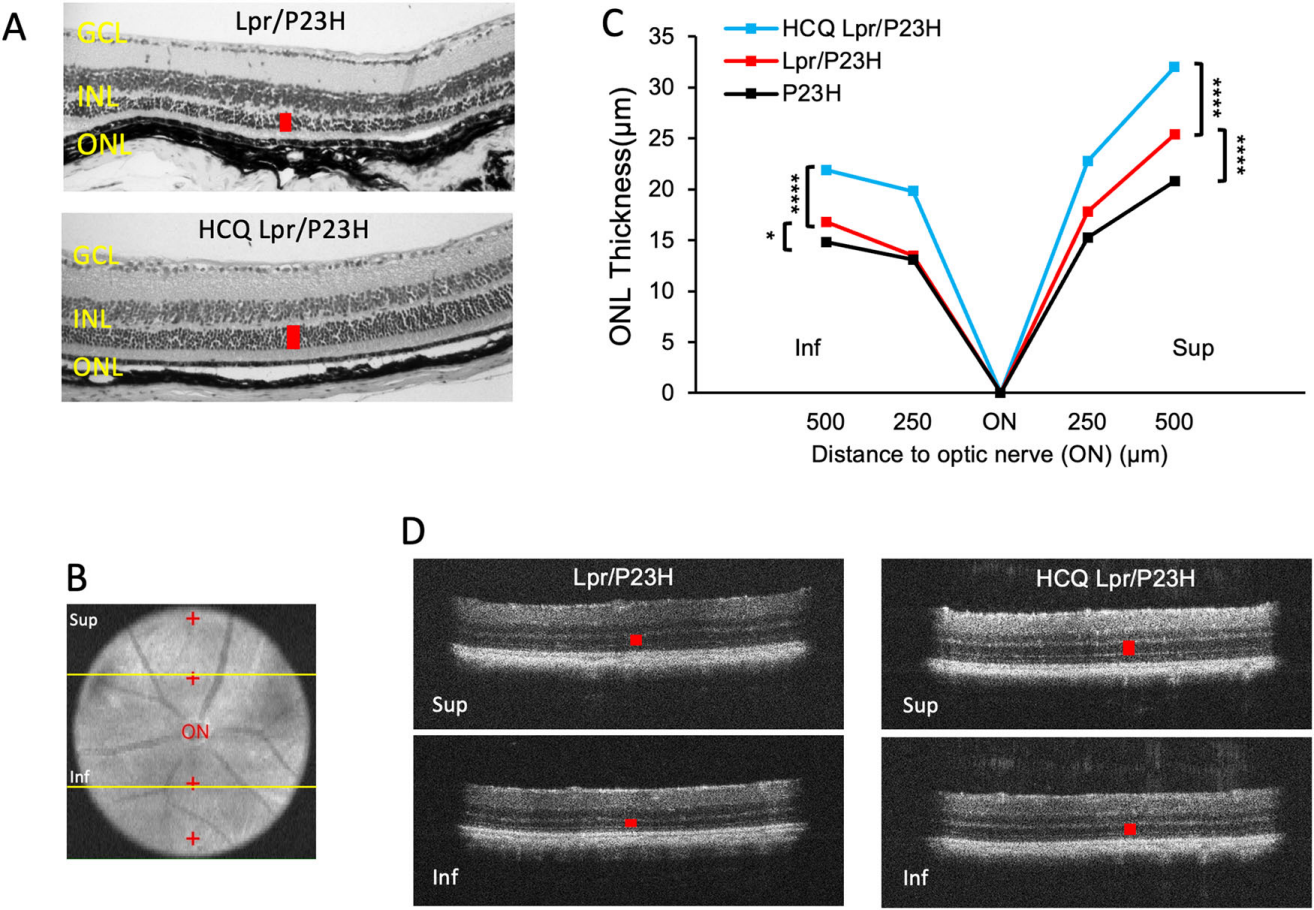
HCQ treatment preserved ONL thickness. **A** Representative H&E staining images show preserved outer nuclear layer thickness (red bar) in the retina of HCQ-treated Lpr/P23H and untreated Lpr/P23H control mice. **B** Representative fundus photo showing the measurement points (red cross) in the superior and inferior of the retina by OCT. **C** Quantification of the thickness of the ONL (indicated by red cross in **B**) of the superior and inferior retina measured at both 250 and 500 µm from the optic nerve head by OCT in HCQ treated Lpr/P23H, Lpr/P23H, and P23H mice at the age of 4 months (*n* = 16 for HCQ Lpr/P23H and Lpr/P23H and *n* = 12 for P23H). **D** Representative OCT images of superior (Sup) and inferior (Inf) retina (indicated by yellow lines in B) of 4-month-old HCQ treated Lpr/P23H and Lpr/P23H controls. *P < 0.05; ****P < 0.0001, 1-way ANOVA. GCL ganglion cell layer, INL inner nuclear layer, ONL.

Histological analysis of retinal samples at 4 months of age revealed that HCQ-treated Lpr/P23H mice maintained a thicker outer nuclear layer (ONL) (Fig. 2A). In P23H mice, PR loss occurs more rapidly in the inferior retina compared to the superior retina [17]. The thickness of the ONL, where PR cell nuclei reside, was measured in vivo using OCT in both the superior and inferior regions of the retina (Fig. 2B–D). Fas deficiency resulted in the preservation of ONL thickness in both the superior and inferior retina in Lpr/P23H mice compared to P23H controls at 4 months of age. Additionally, reducing autophagy flux with HCQ further preserved ONL thickness in Lpr/P23H mice.

### HCQ treatment decreases immune cell activation and levels of inflammatory cytokines in the retinas of Lpr/P23H mice

**Fig. 3:**
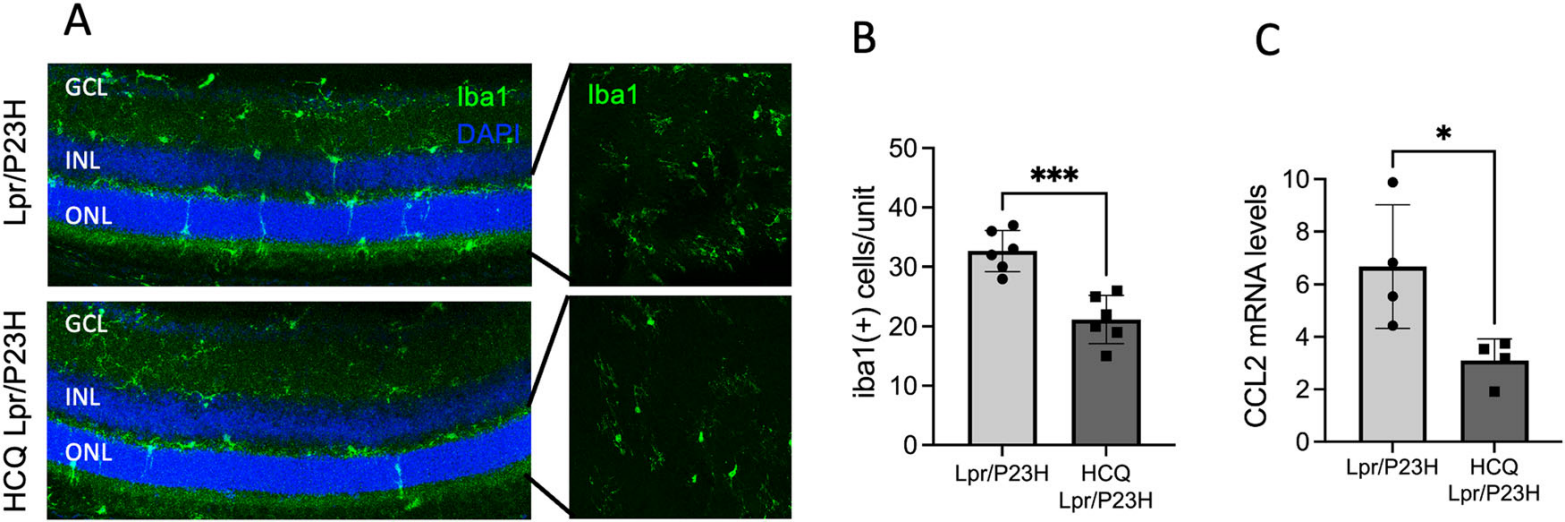
Decreased inflammation in the HCQ-treated Lpr/P23H mice. **A** Representative images of the inferior area of the retinal whole mount and 30 µm retinal sections of HCQ-treated Lpr/P23H and untreated Lpr/P23H mice stained with Iba1 at 1 month of age. **B** Quantification of Iba1-positive cells in the ONL and subretinal space of the inferior retina of HCQ-treated Lpr/P23H and untreated Lpr/P23H mice (*n* = 6) paired *t*-test. **C** mRNA levels of inflammatory cytokines CCL2 in the retinas of HCQ-treated Lpr/P23H, untreated Lpr/P23H normalized to age-matched C57 mice at 1 month of age (*n* = 4) *t*-test and one-way ANOVA. *P < 0.05, ***P < 0.001. GCL gangling, INL inner nuclear layer, ONL outer nuclear layer.

It has been demonstrated that an inflammatory microenvironment plays a crucial role in the progression of retinal diseases. This is evidenced by the upregulation of inflammatory cytokines [23–29, 37–39], which in turn activate and recruit microglia, macrophages, and other immune cells to the retina. To identify immune cells within the retina, we performed Iba1 staining on both retinal whole mounts and cross sections. We observed increased activation and migration of Iba1-positive cells into the ONL in Lpr/P23H mice. Conversely, in HCQ-treated retinas, significantly fewer Iba1-positive cells were detected in the ONL. (Fig. 3A and B). The transcript level of inflammatory cytokine CCL2 was decreased in the retinas of HCQ-treated Lpr/P23H mice as compared to untreated controls (Fig. 3C).

### HCQ treatment improves retinal function in the Lpr/P23H mouse retina

**Fig. 4:**
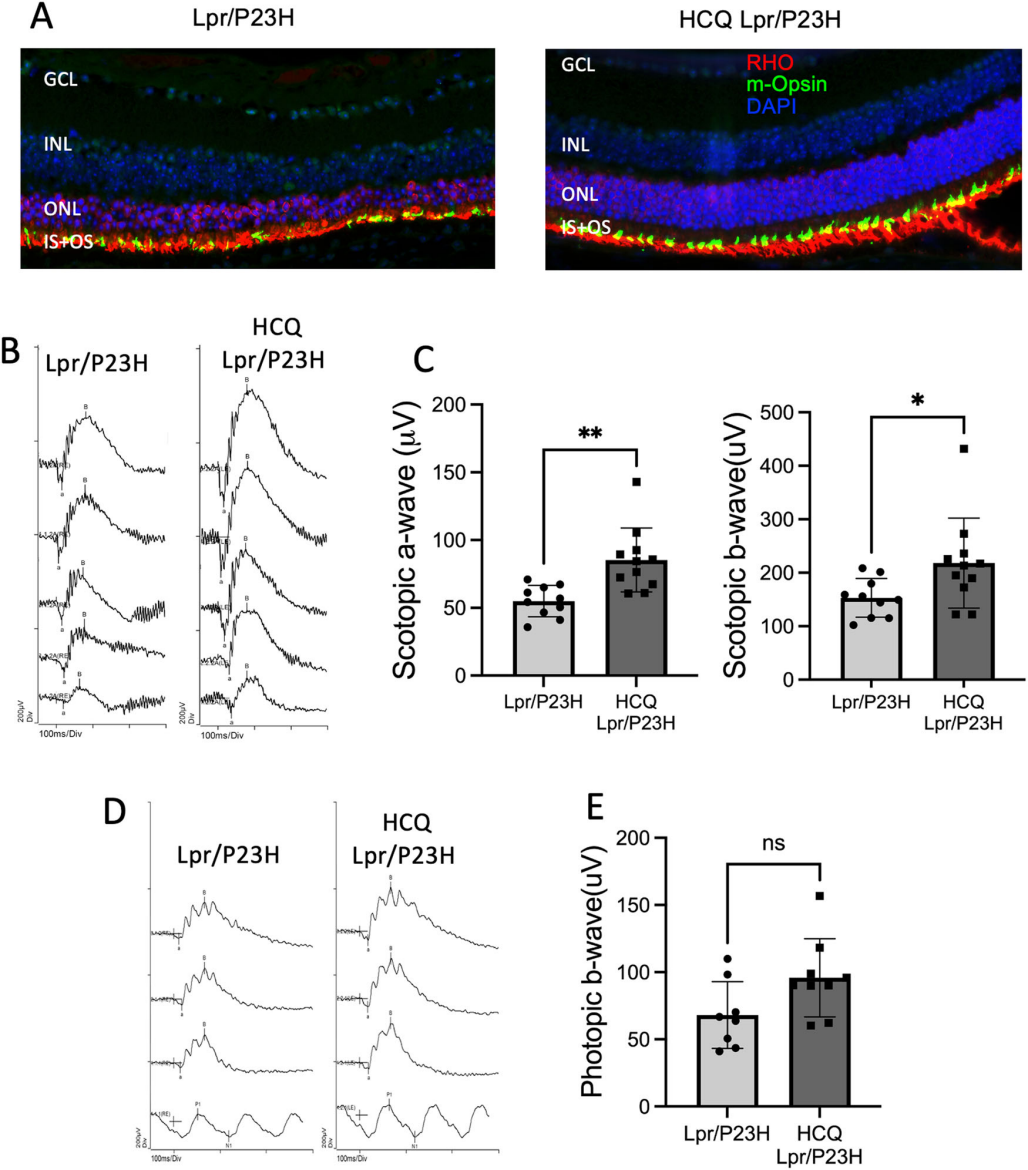
HCQ treatment improved retinal function in Lpr/P23H mice. **A** Representative immunostaining images of the retina of HCQ Lpr/P23H, Lpr/P23H, and C57 mice at 4 months of age, stained with rhodopsin (RHO in red), m-opsin (green), and DAPI (blue). **B** Representative scotopic traces (at 0.01, 1, 10, 32, and 64 cd s/m^2^) and **C** quantification of amplitudes of scotopic a-wave and b-wave of HCQ treated Lpr/P23H (*n* = 11), untreated Lpr/P23H mice (*n* = 10) at 4 months of age. **D** Representative photopic ERG traces (at 10, 32, and 100 cd s/m^2^) and **E** quantification of amplitudes of photopic b-wave of HCQ treated Lpr/P23H and control Lpr/P23H mice at 4 months of age. ns, not significant, *P < 0.05; **P < 0.01; one-tailed Wilcoxon test. GCL ganglion cell layer, INL inner nuclear layer, ONL outer nuclear layer, IS inner segment, OS outer segment.

Preservation of the photoreceptors in the Lpr/P23H by HCQ treatment was further supported by immunostaining of the PR proteins, rhodopsin, and cone-opsin, on retinal sections (Fig. 4A). Visual function of the retina was assessed in HCQ-treated Lpr/P23H and untreated Lpr/P23H control mice using electroretinography (ERG) at 4 months of age. We observed that both scotopic a-wave and b-wave were higher in HCQ-treated Lpr/P23H mice than in age-matched untested Lpr/P23H controls. (Fig. 4B and C). This improved rod ERG responses in the HCQ-treated group as compared to the untreated group, were consistent with the increased immunostaining for the rod-specific protein, rhodopsin, in the retinas of HCQ-treated Lpr/P23H mice. We also observed a trend of increase in photopic ERG response in the treated group, but the difference was not statistically significant (Fig. 4D and E).

### Combined pharmacological inhibition of Fas and autophagy pathways confers PR protection in the P23H mouse retina

**Fig. 5:**
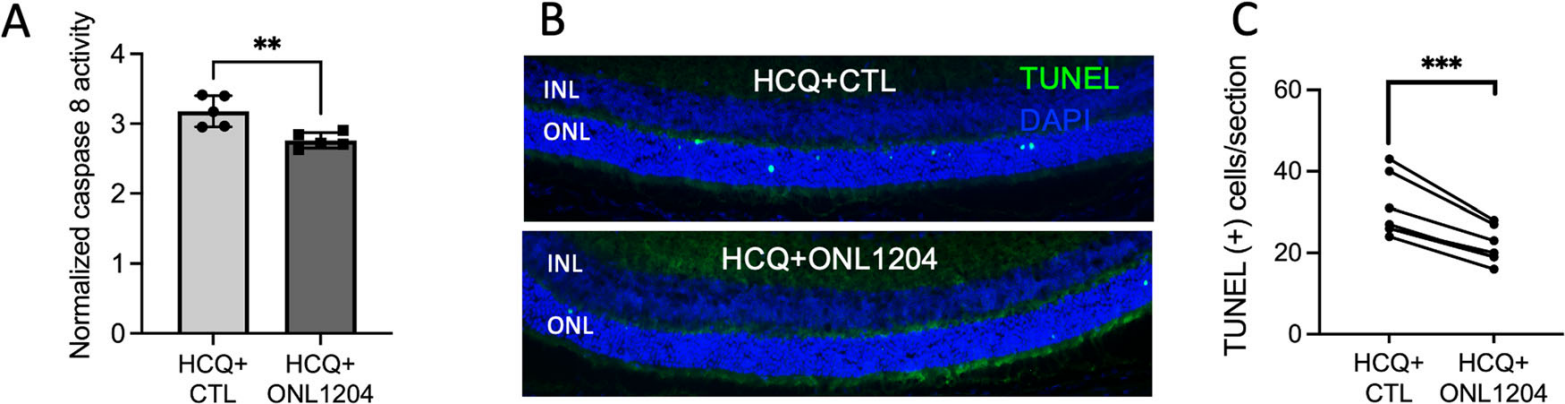
In P23H mice, combining HCQ treatment with intravitreal injections of ONL1204 reduced photoreceptor death than HCQ treatment alone. **A** Quantification of caspase 8 activity in the retina of HCQ + ONL1204 treated and HCQ+ Vehicle-treated eyes of the P23H mice at 1 month of age, normalized to ONL thickness (*n* = 10, 2 retinas from 2 mice were pooled for each sample). **B** Representative TUNEL staining images and **C** quantification of TUNEL-positive cells in the ONL for HCQ + ONL1204 treated and HCQ+ Vehicle-treated P23H eyes at 1 month (*n* = 6). **P < 0.01; ***P < 0.001, *t*-test. INL inner nuclear layer, ONL outer nuclear layer.

**Fig. 6:**
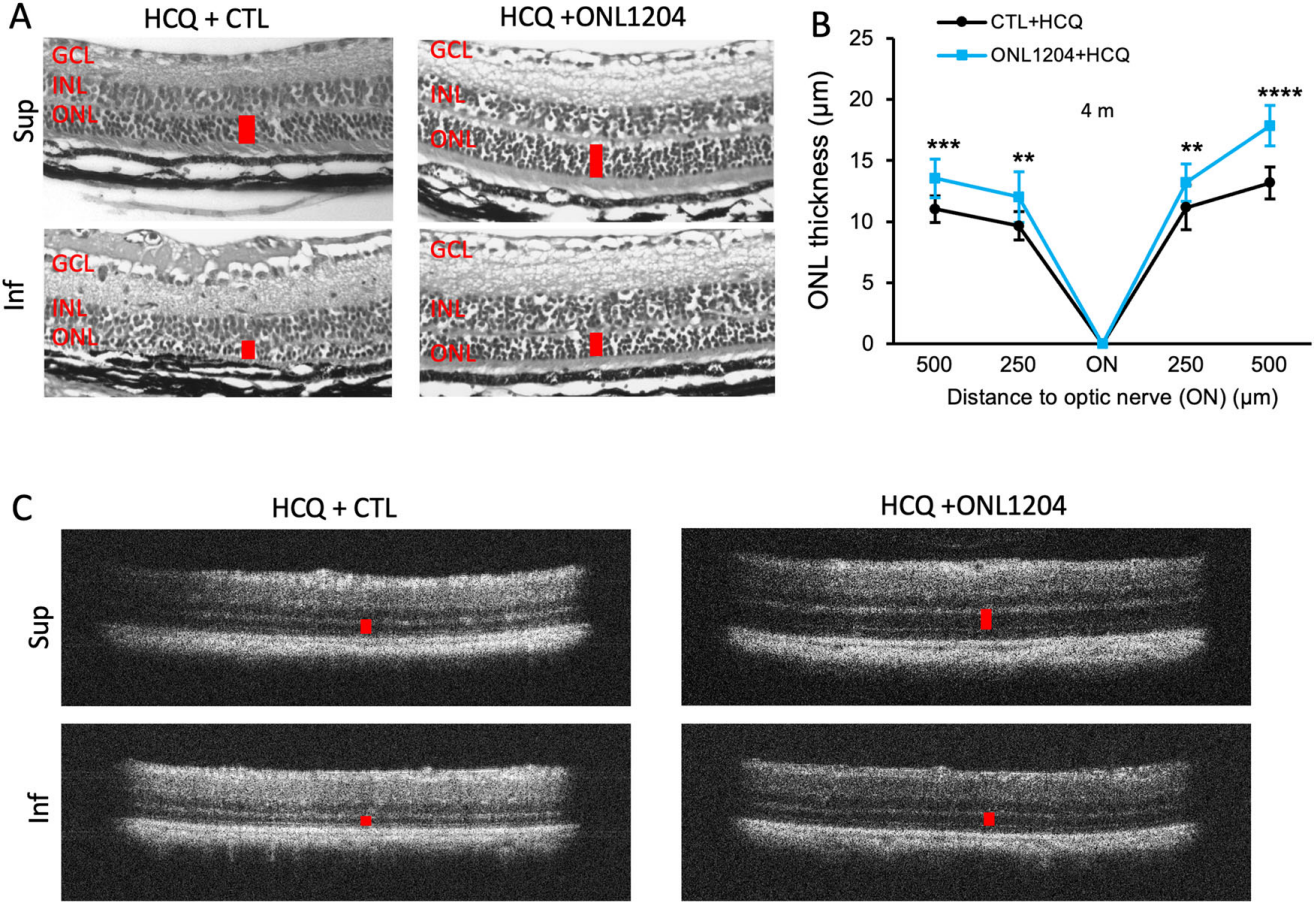
Combination of HCQ with ONL1204 preserved photoreceptors in the retinas of P23H mice. **A** Representative H&E staining images show preserved ONL thickness (red bar) in the retinas of HCQ + ONL1204-treated eyes at 4 months of age. **B** Quantification of the thickness of the ONL of the superior and inferior retina measured at both 250 and 500 µm from the optic nerve head by OCT in HCQ + ONL1204 treated eyes and HCQ + CTL vehicle-treated control eyes at the age of 4 months (*n* = 15). **C** Representative OCT images of superior (Sup) and inferior (Inf) retina of 4-month-old HCQ + ONL1204 treated eye and HCQ + CTL vehicle-treated fellow eyes. **P < 0.01; ***P < 0.001; ****P < 0.0001, unpaired *t*-test. GCL ganglion cell layer, INL inner nuclear layer, ONL outer nuclear layer.

With the proof-of-concept study supporting our hypothesis, we then wanted to assess whether combining the pharmacological reduction of autophagy with pharmacologic inhibition of the Fas receptor can recapitulate the protection conferred by the HCQ treatment in the mouse retina with a genetically defective Fas receptor. This dual therapy was achieved by combining HCQ treatment with intravitreal injections of ONL1204, a small peptide inhibitor of the Fas receptor [32]. In addition to HCQ treatment through drinking water starting from age P21, P23H mice received a 1 µL intravitreal injection of ONL1204 at both ages P14 and 2 months. The fellow eyes received injections of vehicle solution as a control. At 1 month of age, caspase 8 activity was decreased in the retinas of HCQ + ONL1204-treated P23H eyes compared with control vehicle-injected fellow eyes (Fig. 5A). A decreased number of TUNEL-positive PRs was also observed in the retinas of ONL1204 treated eyes as compared to control vehicle-injected fellow eyes at 1 month of age (Fig. 5B and C). Histological analysis of retinal samples at 4 months of age confirmed preserved ONL thickness in the HCQ + ONL1204 treated eyes of the P23H mice compared with vehicle-treated fellow eyes in both the superior and inferior aspects of the retina (Fig. 6A). Thickness of ONL measured in vivo with OCT demonstrated increased thickness in HCQ + ONL treated eyes as compared to control (Fig. 6B and C).

**Fig. 7:**
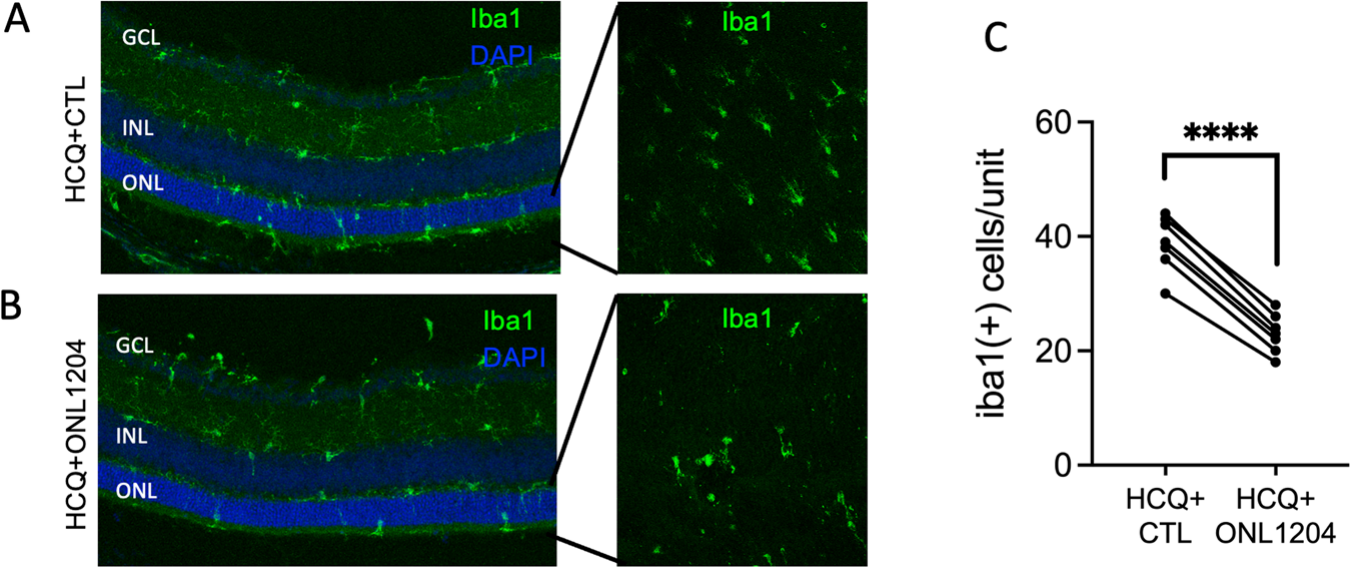
Treatment of HCQ + ONL1204 reduced inflammation in the retinas of P23H mice. **A** Representative immunostaining images of retinal sections and retinal whole mount from inferior retinas of HCQ + ONL1204 treated eyes and **B** HCQ + CTL treated fellow eyes at 1 month of age stained with Iba-1 and DAPI. **C** Quantification of Iba1-positive cells in the ONL and subretinal space of the inferior retina of HCQ + ONL1204 treated eyes and HCQ + CTL treated eyes at 1 month of age (*n* = 7). ****P < 0.0001. Paired *t*-test. GCL ganglion cell layer, INL inner nuclear layer, ONL outer nuclear layer.

Staining for Iba1 on both retinal sections and retinal whole mount (Fig. 7A–C) revealed significantly fewer immune cells in the ONL of the ONL1204-treated P23H mice. These results suggest that combined inhibitions of both autophagy and Fas pathways by HCQ and ONL1204 further improved photoreceptor survival and reduced activation of immune cells in the P23H retinas than autophagy inhibition alone by HCQ.

**Fig. 8:**
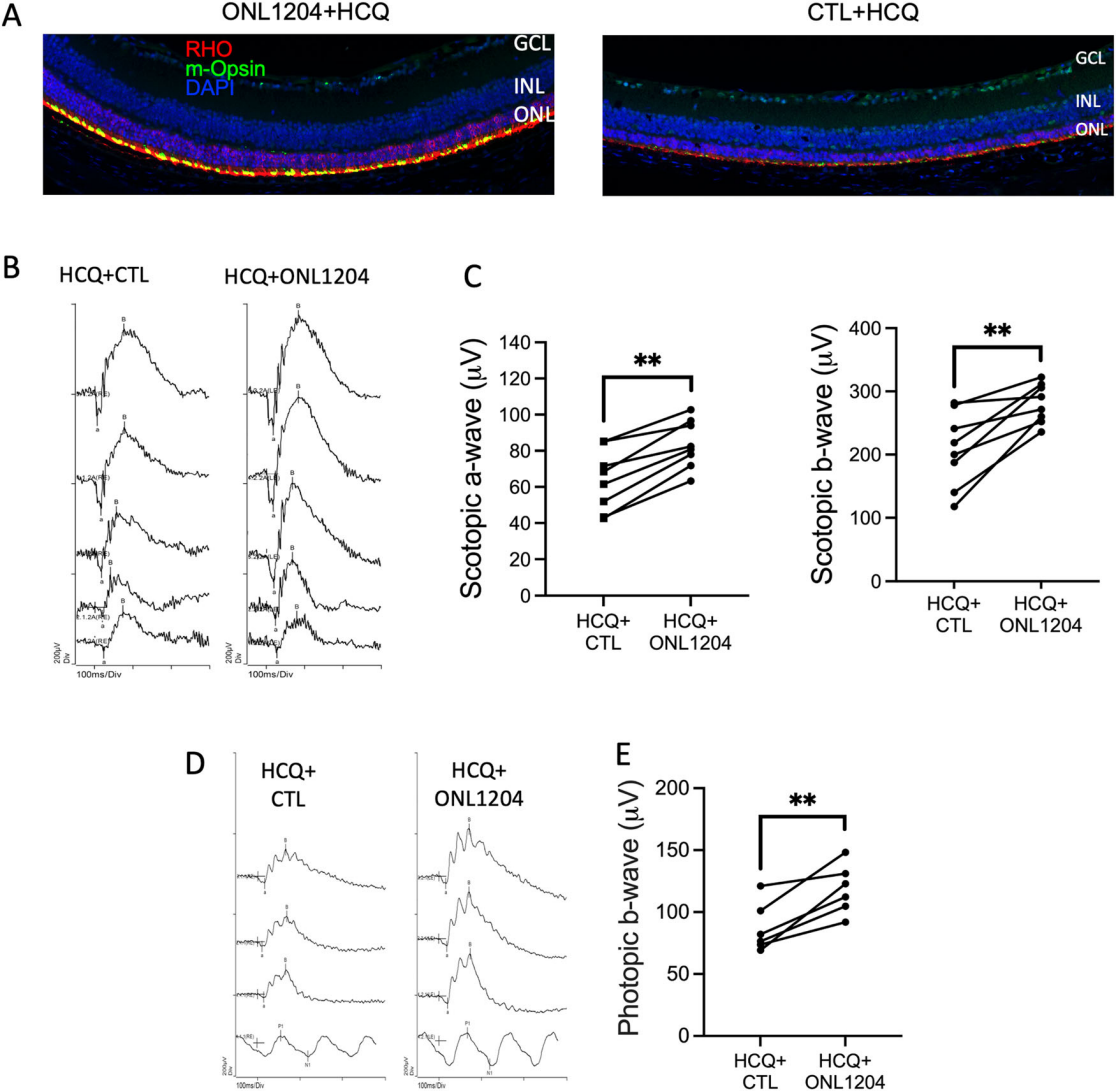
HCQ + ONL1204 treatment preserved visual function in P23H mice. **A** Representative immunostaining images of the inferior retina of HCQ + ONL1204 treated eyes and HCQ+ control vehicle-treated fellow eyes of the P23H mice, at 4 months of age, stained with rhodopsin (red), m-opsin (green), and DAPI (blue). **B** Representative scotopic traces (at 0.01, 1, 10, 32, and 64 cd s/m^2^) and **C** quantification of amplitudes of scotopic a-wave and b-wave of HCQ + ONL1204 treated and HCQ + CTL treated fellow eye at 4 months of age. **D** Representative photopic ERG traces (at 10, 32, and 100 cd s/m^2^) and **E** quantification of amplitudes of photopic b-wave of HCQ + ONL1204 and HCQ + CTL-treated fellow eyes of P23H mice at 4 months of age (*n* = 8), **P < 0.01; one-tailed Wilcoxon test. GCL ganglion cell layer, INL inner nuclear layer, ONL outer nuclear layer.

As was the case for HCQ treatment in the Lpr/P23H mouse, immunostaining showed increased staining for rhodopsin and cone m-opsin in the retinas of HCQ + ONL1204-treated eyes of P23H mice at 4 months of age (Fig. 8A). Consistent with the increased immunostaining, both scotopic and photopic ERG responses of HCQ + ONL1204-treated eyes were significantly higher than the vehicle-treated fellow eyes (Fig. 8B–E). These results indicate that pharmacological inhibition of both autophagy and Fas pathways by combining HCQ and ONL1204 further preserves photoreceptor survival and visual function in the P23H mice than HCQ alone.

## Discussion

A major unmet medical need in treating patients with IRD is a mutation-agnostic therapy for preventing retinal cell death. The extreme heterogeneity in the underlying causative genetic defect makes it very challenging to develop specific therapies for all individual mutations. Previous studies with gene-independent approaches have shown promising protective effects in IRD models. Small peptides derived from the neurotrophic region of pigment epithelium-derived factor (PEDF) given through eye drops prevented photoreceptor death in rd10 mice. That study also showed that PEDF peptides promoted PR survival in the P23H mouse when delivered either through eye drops (short term) or through AAV-mediated gene therapy (long term) [40]. Studies on Photoregulin1, a small molecule modulator of the rod transcription factor Nr2e3, which plays a major role in regulating the development and maintenance of photoreceptors, also showed PR protection in P23H mice [41, 42].

Autophagy and the Fas pathway are interconnected processes in cells. Studies have highlighted the intricate link between these two important signaling pathways during cell death in various pathological conditions during cell death where they can interact synergistically or in opposition [43–48]. Autophagy can regulate caspase-8 by inhibiting its activation or by promoting its degradation [45]. Autophagic protein Beclin-1 can interact with Fas, FasL, and caspase-8 [46]. Targeting both two pathways can be a novel therapeutic approach to various disease conditions. In our previous work, we have shown that blocking Fas receptor activity improves PR structure and function in the P23H and the rd10 mouse models of IRD. In the P23H mouse model, we also showed that hyperactivation of autophagy contributes to PR cell death and that reducing autophagy flux improves PR survival. Given the multiple molecular pathways that contribute to PR degeneration, it seems plausible that combination therapy may provide improved retinal cell survival compared to any one therapy alone. In this work, we have demonstrated that inhibiting the Fas receptor and reducing autophagy flux has a synergistic effect in the P23H mouse retina.

In this study, we used HCQ to reduce autophagy flux. For Fas receptor inhibition, we first used a genetic model (the Lpr mouse) but then extended the findings to the pharmacologic inhibitor of the Fas receptor known as ONL1204. This compound is being developed by ONL Therapeutics (Ann Arbor, MI), and is being studied as a neuroprotective agent in multiple indications, including retinal detachment, open-angle glaucoma, and age-related macular degeneration [23, 28, 30]. This study is the first to use ONL1204 in conjunction with a second agent to deliver an additive neuroprotective effect. As hypothesized, combining HCQ treatment with Fas inhibition, either genetically or pharmacologically, resulted in decreased PR cell loss, reduced inflammation, and improved visual function when compared with HCQ treatment or Fas inhibition alone. The results indicate that simultaneously inhibiting these two key pathways slows the progression of retinal degeneration and improves visual function in the P23H mouse retina.

A major consideration in the interpretation of our results is the generalizability to other forms of IRD beyond the P23H form of adRP. In our previous work, we had shown that the misfolded P23H rhodopsin induces a state of hyperautophagy with a resultant increase in PR cell death. The extent to which hyperautophagy contributes to PR death in other forms of IRD, especially those not caused by mutations in rhodopsin, still remains to be determined. However, our results do confirm the potential for combination therapy. The same can be said for the role of Fas activation in PR death in other forms of IRD besides P23H-Rho. We have shown that Fas-mediated cell death plays a role in PR degeneration in the rd10 mouse, thus suggesting that Fas may be a core common pathophysiologic pathway contributing to retinal degeneration in IRD. However, this needs to be verified in mouse models of IRD secondary to other causative mutations.

Implementation of combination therapies in patients with IRD could face potential regulatory hurdles. The safety of each agent would need to be assessed individually, and consideration would need to be given to any potential interaction between the different therapies. In the case of HCQ, it is already approved for numerous conditions; however, its use in IRD would be off-label. In the case of HCQ, there is also the known potential for retinal toxicity. Though there are guidelines for HCQ dosing and the development of retinopathy [49], it is not known if they would apply to patients with IRD. As for ONL1204, it is currently in clinical development across a number of ophthalmic indications (NCT04744662, NCT05730218, NCT06659445, NCT03780972), in which its safety and efficacy is being evaluated. Use in IRD patients would require further study.

In summary, the work described here demonstrates the potential for combination therapy targeting different pathways to reduce photoreceptor cell death in improve retinal function in patients with IRD. Additional work is warranted to help bring these potential therapeutics to patients.

## Data Availability

All data generated and analyzed in this study are presented in this published article. Primary data may be made available from the corresponding author upon reasonable request.
